# Successful treatment outcome of primary melioidosis pneumonia-a case report from Bangladesh

**DOI:** 10.1186/s13104-016-1910-0

**Published:** 2016-02-16

**Authors:** Samira Rahat Afroze, Md. Raziur Rahman, Lovely Barai, Md. Delwar Hossain, Khwaja Nazim Uddin

**Affiliations:** Department of Internal Medicine, BIRDEM General Hospital and Ibrahim Medical College, Dhaka, Bangladesh; Microbiology Department, BIRDEM General Hospital, Dhaka, Bangladesh

**Keywords:** Bangladesh, *Burkholderia pseudomallei*, Melioidosis, Pneumonia

## Abstract

**Background:**

Melioidosis is endemic in tropical Australia and Southeast Asian countries and its causative organism *Burkholderia pseudomallei* is a recognized cause of pneumonia in these regions. Recent isolation of the organism in the soil of Kapasia, Gazipur, Bangladesh has proven its exposure among the population residing in endemic areas of our country. Pneumonia is the most common presentation of melioidosis. Acute, subacute and chronic pneumonia due to *B. pseudomallei* can present as primary or secondary pneumonia. Treatment of such cases are challenging as well. Till date, few cases of acute and chronic pneumonia due to melioidosis occurring in local Bangladeshis as well as in returning travelers to Europe have been reported. To the best of our knowledge, this is the first reported case of primary melioidosis pneumonia declared cured after a 27 weeks of treatment regimen from Bangladesh.

**Case presentation:**

A 43-year-old Bangladeshi gentleman, known diabetic, hypertensive, smoker, presented with the complaints of recurrent episodes of low to high grade intermittent fever, productive cough with occasional haemoptysis and 10 kg weight loss over one and half months. Poorly responding to conventional antibiotics, he was suspected as a case of pulmonary tuberculosis. Examination and investigations revealed left sided consolidation with cavitary lesion, hepato-splenomegaly and sputum analysis confirmed growth of *Burkholderia pseudomallei*. The patient was successfully treated as a case of primary melioidosis pneumonia.

**Conclusion:**

Often misdiagnosed and empirically treated as tuberculosis, untreated melioidosis pneumonia may even lead to death. Therefore, melioidosis should be suspected in appropriate clinical scenario in patients with a history of residing in or traveling to endemic areas. In Bangladesh, time has come to explore whether melioidosis should be considered as an emerging infectious disease.

## Background

*Burkholderia pseudomallei*, a gram-negative bacillus responsible for the infectious disease melioidosis, is a common soil and fresh water saprophyte in tropical and subtropical regions between latitudes 20°N and 20°S [1]. Although endemic in tropical Australia and in Southeast Asian countries where it is recognized as a public health problem [2], the increasing number of reported cases in Bangladesh [3] and India [1] are alarming. In Bangladesh, this organism has already been isolated from the soil of Kapasia,.Gazipur in 2013 [4], rendering this nation as a definite country for melioidosis. A recent study has also identified serological evidence to *B. pseudomallei* infection and its exposure being relatively common in local Bangladeshis [5]. Pneumonia in both acute and chronic forms are recognized as a mode of presentation of this disease and treatment of such cases are challenging. This case report highlights the successful treatment outcome in a diabetic male suffering from primary melioidosis pneumonia.

## Case presentation

A 43-year-old Bangladeshi man, known hypertensive and a smoker (18 pack-year), was admitted under Internal Medicine Department of Bangladesh Institute of Research and Rehabilitation in Diabetes, Endocrine and Metabolic Disorders (BIRDEM) General Hospital, with the complaints of recurrent episodes of fever and cough for one and half months. Fever was intermittent, initially low grade, later became high grade (maximum recorded 104 °F) which subsided with sweating, not associated with chills and rigor. His cough which was occasional and dry at first, later became more frequent, productive, and without chest pain, dyspnoea or leg swelling. Sputum was mucoid without any foul smell.

There were no history or features suggestive of chronic obstructive pulmonary disease, bronchial asthma and sinusitis. Previous tuberculosis or contact with known case was denied. He was a returning worker from Brunei, where he had been working as a carpenter since 2008. He had visited his home district Bhramanbaria, Bangladesh, four and half months prior to his illness.

During this period of illness he had lost 10 kg weight. He was treated in Brunei three times with oral antibiotics and antipyretics without much improvement. He was diagnosed as diabetes mellitus on his 3rd follow-up visit in Brunei and was treated with tablet glicazide. By that time he also developed occasional mild haemoptysis. Suspecting pulmonary tuberculosis, he was sent back to Bangladesh. On the day he returned from Brunei, he took admission in BIRDEM General Hospital for thorough.evaluation and management.

At the time of admission he had high grade fever, productive cough (whitish sputum). On examination he looked ill, temperature was 102 °F, had oral candidiasis, was dehydrated without clubbing or lymphadenopathy. Features of consolidation in his left lung and palpable, non-tender hepato-splenomegaly (liver 2 cm, firm; spleen 4 cm from respective costal margins) were present. Other systemic findings were unremarkable.

**Fig. 1:**
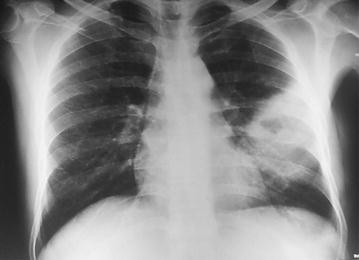
Chest x-ray posterior–anterior view on admission (04/09/2014) showing homogenous opacity with a cavitary lesion occupying parts of left middle and lower zones

**Fig. 2:**
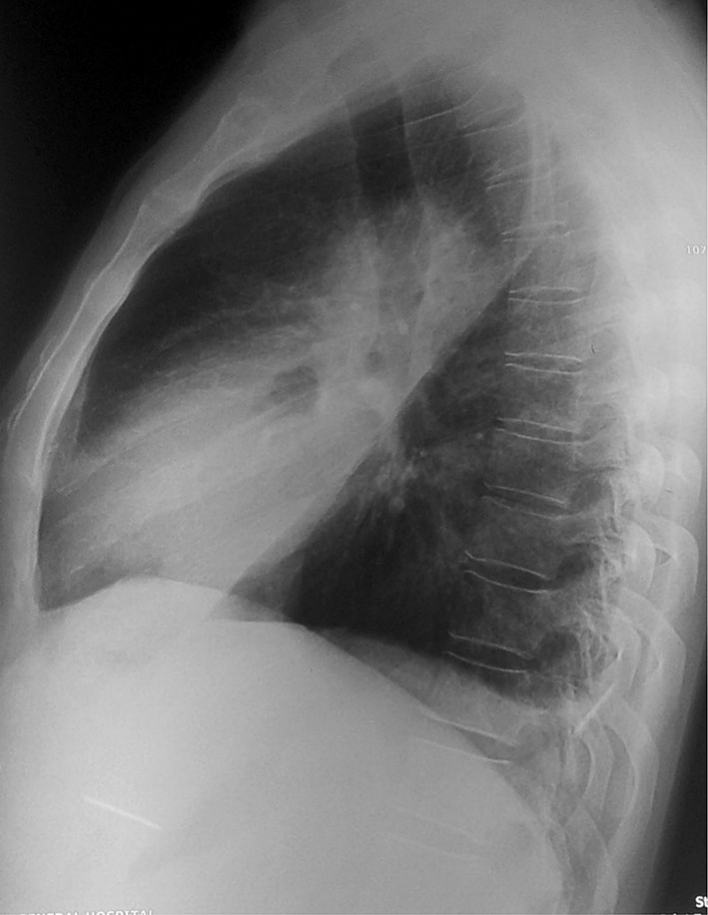
Plain x-ray of chest, left lateral view (on admission)

Reports of initial investigations done in Brunei were unavailable. During his stay at our hospital, investigations revealed a high erythrocyte sedimentation rate (ESR, 150 mm in 1st hour) despite a normal complete blood count. His blood sugar profile (random blood sugar 16.2 mmol/L, glycated hemoglobin 12.6 %) was uncontrolled. Chest x-ray posterior-anterior view showed homogenous opacity with a cavitary lesion occupying parts of left middle and lower zones (Figs. 1, 2). Ultrasonography of whole abdomen reported mildly enlarged liver with fatty change (Grade II) and enlarged spleen (16 cm) with left sided mild pleural effusion.

**Fig. 3:**
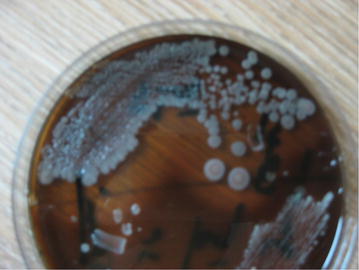
Photograph showing growth of *Burkholderia pseudomallei* in Blood agar

Sputum analysis revealed presence of plenty pus cells, gram negative bacteria on Gram’s stain and growth of *B. pseudomallei* (Fig. 3) which was sensitive to augmentin, aztroenam, cefixime, ceftriaxone, ceftazidime, cefotaxime, ciprofloxacin, co-trimoxazole, imipenem and piperacillin-tazobactum. Sputum polymerase chain reaction (PCR) for *B. pseudomallei* was also positive. Sputum for malignant cell, acid fast bacilli (2 samples), GeneXpert Mycobacterium tuberculosis/rifampicin (MTB/RIF) were not detected and Montoux test was negative. Blood and urine cultures revealed no growth. Tripple antigen, liver function tests, serum creatinine, lipid profile were normal.

His high blood sugar level was controlled with subcutaneous premixed insulin. For melioidosis, he was treated with ceftazidime 2 g intravenous 8 h for 3 weeks followed by maitainance therapy with capsule doxycycline (100 mg twice daily) and tablet cotrimoxazole (160 + 800 mg, twice daily) for 6 months. He was also advised to quit smoking. He responded well to the treatment. His fever subsided after 13 days, cough became unproductive after 20 days and subsided after 27 days.

**Fig. 4:**
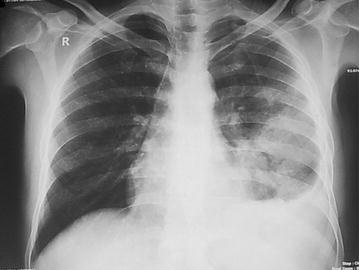
Chest x-ray posterior–anterior view after 2 weeks of treatment showing radiological improvement

On his first follow up visit at 2 weeks the patient was feeling better. Radiological improvement was evidenced by decreasing lung opacity on chest x-ray posterior-anterior view (Fig. 4) and normal size of liver and spleen on ultrasonography of whole abdomen. Left sided pleural effusion was detected by both chest x-ray and ultrasonography.

**Fig. 5:**
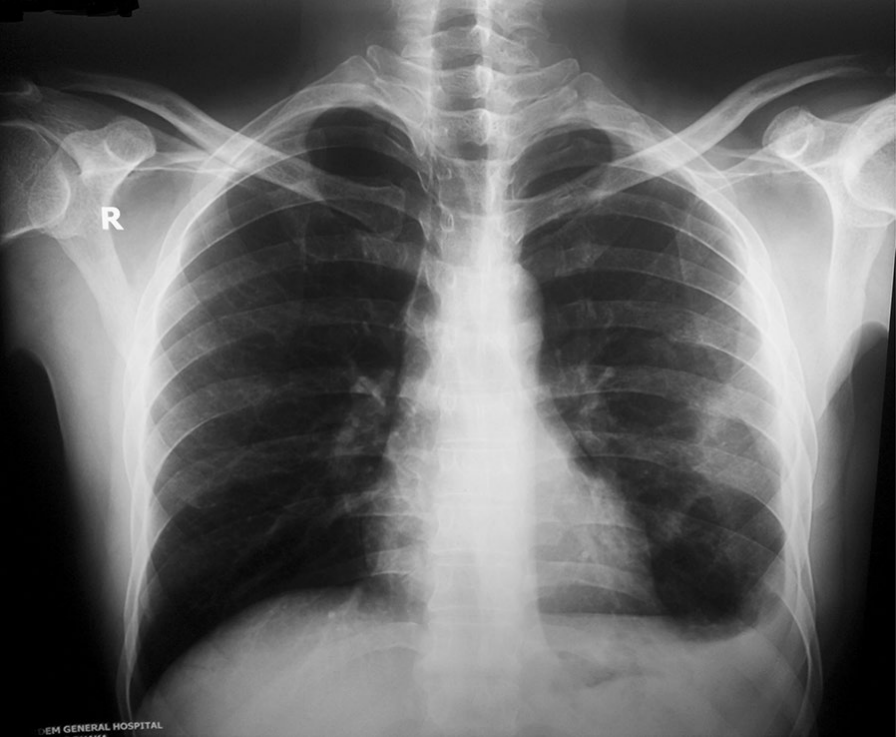
Second follow-up chest x-ray posterior–anterior view after 6 weeks of treatment showing significant radiological improvement with decreased pleural effusion (*left*)

Second follow up visit at 6 weeks demonstrated further symptomatic improvement including 2 kg weight gain. There was significant reduction of the lesion in his left lung radiologically (Fig. 5) and ESR was decreasing (54 mm in 1st hour). Two days after this visit, the patient went back to Brunei to continue his job there and was advised for a follow up visit on 6th April 2015 with reports.

At the time of his final follow up at 6 months, although the patient was unable to return home, the advised follow-up investigations were done in Brunei and were sent by email. Reports revealed a normal complete blood count and ESR (2 mm in 1st hour). Chest x-ray posterior–anterior view reported ‘no active lung lesion’. At this stage, treatment was stopped and the patient was declared cured of melioidosis after 27 weeks of treatment.

## Discussion

Pneumonia is the most common presentation of melioidosis occurring in approximately half of all cases. It is thought that lung involvement arises after hematogenous spread following inoculation [2]. Higher rainfall is significantly associated with sepsis and pneumonia suggesting that environmental conditions during the monsoon season may be associated with inhalation rather than inoculation as the primary mode of acquisition [6]. *B. pseudomallei* strains differ in their individual ability to cause disease, the outcome also clearly depends on the immune status and response of the infected host [1].

Up to 80 % of patients with melioidosis have one or more risk factors for the disease, diabetes mellitus being the most important risk factor (up to 60 % of patients). Other risk factors include heavy alcohol use, chronic pulmonary disease, chronic renal disease, thalassamia, glucocorticoid therapy and cancer [7]. Cheng and Currie [2] in a review stated that patients with diabetes mellitus have a high incidence of melioidosis, with up to 60 % of patients having preexisting or newly diagnosed type 2 diabetes. Case–control and population-based studies in Australia and Thailand gave estimated relative risk of 5.9–13.1. [2] Our patient was diagnosed type 2 diabetic during his course of illness.

Melioidosis pneumonia is a diverse illness that can range from acute, fulminant sepsis with multifocal lung infiltrates to chronic infection that mimics tuberculosis both clinically and radiologically [8]. Melioidosis pneumonia can be the primary presenting feature, or can develop secondary to initial illness at a distant site, or can develop in patients with bacteremia without an initial evident focus [9]. *B. pseudomallei* may remain latent for many years and then reactivate; however, most melioidosis cases are thought to occur soon after exposure [10].

In a 21-year study in the top end of the Northern Territory, Australia, cases of melioidosis pneumonia were observed. Acute/subacute melioidosis was diagnosed if symptoms had been present for <2 months; chronic melioidosis was diagnosed if symptoms at presentation had been present for ≥2 months. Primary pneumonia was diagnosed when there were clinical and radiologic features of pneumonia present within 48 h of admission and there was no other initial clinical focus evident. Secondary pneumonia was diagnosed when respiratory symptoms and pulmonary infiltrates developed 48 h after admission in patients with an initial non-pulmonary presentation. Among 624 cases of culture-confirmed melioidosis, primary pneumonia, accounted for 51 % of presentations, occurring mostly during the wet season. Nineteen percent cases developed secondary pneumonia during the course of their illness. The majority of primary pneumonia cases were acute/subacute (291 cases, 91 %); chronic disease (28 cases, 9 %) was seen less commonly. Acute/subacute infection was associated with bacteremia, septic shock, and death. While upper-lobe involvement was common in both acute/subacute and chronic pneumonia, lower lobe or multilobar involvement was more common in acute/subacute pneumonia [11].

Haemoptysis is rare in acute disease but may be present in up to 31 % of patients with the chronic form of the disease. Pleural involvement occurs in 9–33 % of cases and thoracic empyema is occasionally seen. A more indolent presentation of melioidosis pneumonia is associated with a better outcome. The chest radiograph in acute disease commonly shows either a localized patch or bilateral diffuse patchy alveolar infiltration or multiple nodular lesions which may coalesce, cavitate (cavities are usually thin-walled and rarely contain air-fluid level) and form abscesses [12, 13].

In the chronic form of the disease, the chest radiograph findings may be difficult to distinguish from that of pulmonary tuberculosis which typically involves the upper lobes with patchy alveolar infiltrates and cavitations. Sparing of the apical region and lack of calcification suggest the likelihood of melioidosis rather than pulmonary tuberculosis [12, 13].

Our patient presented with features of primary pneumonia. He had significant weight loss, occasional haemoptysis and was initially suspected as a case of pulmonary tuberculosis. Among the recognized risk factors [11] for primary melioidosis pneumonia he had a history of diabetes mellitus, smoking and his symptoms started during the wet season. Investigations confirmed melioidosis pneumonia with multilobar lung involvement and cavitary lesions. Besides pneumonia, his hepato-splenomegaly also resolved with treatment for melioidosis. Although he presented within 2 months of his symptoms, it was not possible to say whether it was acute or subacute pneumonia due to lack of clear guidelines.

Till date, 6 cases of melioidosis pneumonia were reported in Bangladeshis among locals and as returning travelers to Europe. Among them three were acute/subacute cases [3, 14, 15] and three were chronic cases [16–18] One case was secondary melioidosis pneumonia [15]. Five cases showed involvement of upper lung zone [3, 14, 16–18]. Cavity lesions were observed in three cases [3, 17, 18]. Mid and lower lung zone involvement was observed in our case, as was seen in another case with acute pneumonia [15]. Out of the six cases, four cases showed improvement with treatment but none of the cases were followed up till a complete cure [14, 15, 17, 18] and two patients failed to respond to treatment and died [3, 16].

However, there remains a debate as from where our patient acquired the primary infection from, Bangladesh or Brunei? Prior to development of his symptoms, he had a history of recent traveling to his home district in Bangladesh, where seropositivity to *B. pseudomallei* has been observed already [5]. On the other hand, Brunei is also well recognized as an endemic country for melioidosis [19, 20] where this patient has been working for the past few years.

To the best of our knowledge, this is the first case report of primary melioidosis pneumonia declared cured after a 27 weeks completed treatment regimen from Bangladesh.

## Conclusion

Melioidosis, if diagnosed early and treated accordingly, can prevent complications, even save a patient’s life, as reported in this article. In countries where tuberculosis is endemic, newly emerging diseases like melioidosis may be overlooked. Suspicion of melioidosis should be considered in appropriate clinical scenario, especially in patients with a history of residing in or traveling to endemic areas. In Bangladesh, isolation of *B. pseudomallei* from the soil, serological evidence of exposure to the organism and increasing numbers of diagnosed melioidosis cases among Bangladeshis are alarming. This warrants in creating an awareness among clinicians and microbiologists in Bangladesh about this organism and the disease caused by it.

## Consent to publish

Written informed consent was obtained from the patient for publication of this case report and any accompanying images. A copy of the written consent is available for review by the Editor-in-Chief of this journal.

## Abbreviations

BIRDEM: Bangladesh Institute of Research and Rehabilitation in Diabetes, Endocrine and Metabolic Disorders
ESR: erythrocyte sedimentation rate
PCR: polymerase chain reaction
MTB/RIF: *Mycobacterium tuberculosis*/rifampicin

## References

[1] Adler NRL, Govan B, Cullinan M, Harper M, Adler B, Boyce JD (2009). The molecular and cellular basis of pathogenesis in melioidosis: how does *Burkholderia pseudomallei* cause disease?. FEMS Microbiol Review..

[2] Cheng AC, Currie BJ (2005). Melioidosis: epidemiology, pathophysiology, and management. Clin Microbiol Rev.

[3] Barai L, Jilani SA, Haq JA (2014). Melioidosis-Case reports and review of cases recorded among Bangladeshi population from 1988–2014. Ibrahim Med Coll J..

[4] News Medical (2013). Melioidosis: deadly bacteria in Gazipur soil of Bangladesh. Bangladesh Med J..

[5] Maude RR, Maude RJ, Ghose A, Amin MR, Islam MB, Ali M (2012). Seroepidemiological surveillance of *Burkholderia pseudomallei* in Bangladesh. Trans R Soc Trop Med Hyg.

[6] Currie BJ, Jacups SP (2003). Intensity of rainfall and severity of melioidosis Australia. Emerg Infect Dis.

[7] Wiersinga WJ, Currie BJ, Sharon J, Peacock SJ (2012). Melioidosis. N Engl J Med.

[8] White NJ (2003). Melioidosis. Lancet.

[9] Currie BJ, Fisher DA, Howard DM (2000). Endemic melioidosis in tropical northern Australia: a 10-year prospective study and review of the literature. Clin Infect Dis.

[10] Currie BJ, Fisher DA, Anstey NM, Jacups SP (2000). Melioidosis: acute and chronic disease, relapse and re-activation. Trans R Soc Trop Med Hyg.

[11] Meumann EM, Cheng AC, Ward L, Currie BJ (2012). Clinical features and epidemiology of melioidosis pneumonia: results from a 21-year study and review of the literature. Clin Infect Dis.

[12] Everett ED, Nelson RA (1975). Pulmonary melioidosis. Observations in thirty-nine cases. Am Rev Respir Dis..

[13] Dhiensiri T, Puapairoj S, Susaengrat W (1988). Pulmonary melioidosis: clinical-radiologic correlation in 183 cases in Northeastern Thailand. Radiology.

[14] Struelens MJ, Mondol G, Bennish M, Dance DAB (1988). Melioidosis in Bangladesh: a case report. Trans R Soc Trop Med Hyg.

[15] Majumer MI, Haque MM, Ahmed MW, Alam MN, Rahman MW, Akter F (2013). Melioidosis in an adult male. Mymensingh Med J..

[16] Kibbler CC, Roberts CM, Ridgway GL, Spiro SG (1991). Melioidosis in a patient from Bangladesh. Postgrad Med J.

[17] Hoque SN, Minassian M, Clipstone S, Loyd-Owen SJL, Sheridan E, Lessing MPA (1999). Melioidosis presenting as septic arthritis in Bengali men in East London. Rheumatology.

[18] Uddin KN, Hossain M, Mansur A, Hoque MJA, Khan AR (2001). Melioidosis-a case report. J Bangladesh Coll Phys Surg..

[19] Kadir KAA, Satyavani M, Pande K (2014). Melioidosis: antibiogram of cases in Brunei Darussalam. Brunei Int Med J..

[20] Pande KC, Kadir KA (2011). Melioidosis of the extremities in Brunei Darussalam. Singapore Med J.

